# The human axial length and choroidal thickness responses to continuous and alternating episodes of myopic and hyperopic blur

**DOI:** 10.1371/journal.pone.0243076

**Published:** 2020-12-02

**Authors:** Samaneh Delshad, Michael John Collins, Scott Andrew Read, Stephen James Vincent

**Affiliations:** Queensland University of Technology (QUT), Centre for Vision and Eye Research, School of Optometry and Vision Science, Institute of Health and Biomedical Innovation, Kelvin Grove, Queensland, Australia; National Yang-Ming University Hospital, TAIWAN

## Abstract

**Purpose:**

To investigate the change in axial length (AxL) and choroidal thickness (ChT) in response to continuous and alternating episodes of monocular myopic and hyperopic defocus.

**Methods:**

The right eye of sixteen young adults was exposed to 60 minute episodes of either continuous or alternating myopic and hyperopic defocus (+3 DS & -3 DS) over six separate days, with the left eye optimally corrected for distance. During alternating defocus conditions, the eye was exposed to either 30 or 15 minute cycles of myopic and hyperopic defocus, with the order of defocus reversed in separate sessions. The AxL and ChT of the right eye were measured before, during and after each defocus condition.

**Results:**

Significant changes in AxL were observed over time, dependent upon the defocus condition (p < 0.0001). In general, AxL exhibited a greater magnitude of change during continuous than alternating defocus conditions. The maximum AxL elongation was +7 ± 7 μm (p = 0.010) in response to continuous hyperopic defocus and the maximum AxL reduction was -8 ± 10 μm of (p = 0.046) in response to continuous myopic defocus. During both 30 and 15 minute cycles of alternating myopic and hyperopic defocus of equal duration, the effect of opposing blur sessions cancelled each other and the AxL was near baseline levels following the final defocus session (mean change from baseline across all alternating defocus conditions was +2 ± 10 μm, p > 0.05). Similar, but smaller magnitude, changes were observed for ChT.

**Conclusions:**

The human eye appears capable of temporal averaging of visual cues from alternating myopic and hyperopic defocus. In the short term, this integration appears to be a cancellation of the effects of the preceding defocus condition of opposite sign.

## Introduction

Optical defocus can lead to predictable changes in choroidal thickness and eye growth in various animal species [1–5]. Rapid choroidal thickening in response to imposed myopic defocus [6, 7] precedes a slowing of longer-term eye growth [8–11], while rapid choroidal thinning in response to imposed hyperopic defocus [6, 7] precedes accelerated eye growth [8–11]. These compensatory changes result in the retinal photoreceptors moving closer to the defocused image plane. In humans, the quality of visual experience can also influence ocular growth. In eyes experiencing chronic deprivation of form vision through ocular conditions such as ptosis [12, 13], congenital cataract [14], corneal opacity [15], and vitreous haemorrhage [16], abnormal ocular growth is often observed. Similarly, manipulation of retinal image focus through optical treatment strategies such as bifocal or multifocal spectacles [17, 18], soft multifocal contact lenses [19, 20], or orthokeratology [21–24] have been linked with reduced progression of axial myopia in children. Investigations in human eyes have also shown a bi-directional response to short-term imposed continuous myopic and hyperopic defocus in children [25] and adults [26–31], with a small magnitude axial length reduction associated with rapid choroidal thickening in response to myopic defocus and axial elongation associated with rapid choroidal thinning in response to hyperopic defocus.

In the absence of ocular pathology leading to a disruption in form vision, the visual control of eye growth could be associated with the dynamics of visual experience and the type and magnitude of defocus to which the eyes are exposed on a daily basis. Natural visual scenes typically comprise objects at varying distances that produce myopic and hyperopic defocus, and well-focused retinal images, depending on where the eyes are fixating and focused within the environment [32–34]. The temporal integration of these defocus signals may provide input to the regulation of eye growth [6, 32, 33, 35]. Studies using various animal models have shown that over a period of days or weeks, the eyes use a complex method for integrating defocus signals over time rather than a simple linear summation of the blur that it experiences [6, 35–44]. These studies suggest that the temporal integration of defocus signals is dependent upon the sign and power of the defocus experienced [6, 36–43], and the frequency and duration of individual episodes of blur [37, 44].

In humans, while studies have shown that the eye is responsive to short-term continuous myopic and hyperopic defocus by changing its axial length and choroidal thickness [25–31], it is not known how the eye responds to more complex temporally varying patterns of defocus, as might be encountered in real-world visual scenes. In this study, we tested the hypothesis that the short-term response of axial length and choroidal thickness to alternating episodes of myopic and hyperopic defocus in the human eye will reflect a simple temporal summation of the blur signals.

## Materials and methods

Sixteen young adults (11 females, 5 males) aged between 19 and 34 years (mean ± SD, 24.8 ± 4.1 years) were recruited for the study. This sample size provided 80% power to detect an intrasession axial length change of 10 μm at the 5% level, assuming a standard deviation of 10 μm [26]. All subjects were free from any systemic or ocular diseases and had no prior history of eye injury or surgery. Before the study, each subject underwent an initial screening to ensure good ocular health and normal binocular vision, and to determine their refractive status. The spherical equivalent refraction across subjects ranged between +0.50 DS and -2.75 DS with a mean ± SD of -0.57 ± 1.12 DS. All subjects had anisometropia of less than 0.50 DS, astigmatism of ≤ -0.75 DC and exhibited visual acuity of at least 0.00 logMAR (20/20 Snellen acuity) in both eyes. No contact lens wearers were included in the study. Ethics approval was obtained from the Queensland University of Technology human research ethics committee prior to commencement of the study. Written informed consent was obtained from all subjects, who were treated in accordance with the Declaration of Helsinki.

This study involved the measurement of axial length before, during and after six different defocus conditions. Subfoveal choroidal thickness was also measured before and after exposure to defocus. Each defocus condition was conducted on a separate day in order to allow any potential effects to dissipate from the prior defocus session (a minimum 24-hour washout period between the defocus conditions). The order of the defocus conditions was randomized between subjects to ensure no order-related bias in the data. To minimize the potential for a confounding interaction between the diurnal variations in axial length and subfoveal choroidal thickness [45, 46] and the eye’s response to defocus [47], all measurement sessions were conducted at a similar time of day (between 8:00 am and 2:00 pm with an average daily time difference of 62 ± 23 minutes between sessions within each subject), and at least two hours after the subjects’ reported time of waking. None of the subjects enrolled in this study were taking any medications which could influence their ocular biometry measurements. Since smoking may affect the measurement of choroidal thickness, smokers were also not included in this study [48]. Due to the influence of caffeine intake and dynamic exercise on ocular biometry, all participants were asked to abstain from consumption of caffeine or performing vigorous exercise for at least 1 hour prior to the start of each measurement session [49, 50]. Since prior visual tasks (e.g. intense near-work) could potentially affect measurements of axial length and choroidal thickness, each subject completed a 20 minute “washout period” during which they watched a movie of their choosing at a 6 m distance with their optimal distance refractive correction before each measurement session. Following completion of the “washout period”, baseline measurements of axial length and subfoveal choroidal thickness were obtained from the right eye, and then, a 60 minute monocular “defocus period” was conducted.

**Fig 1:**
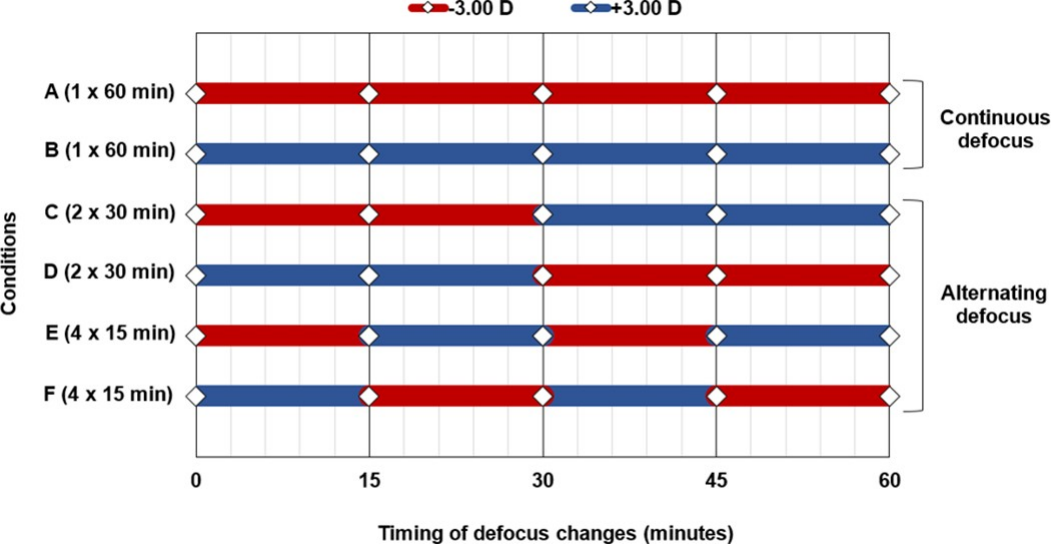
Illustration of the type of defocus imposed on the right eye for each measurement day. On six separate measurement days, the right eye was exposed to one of the following conditions (in a randomized order): (A) continuous hyperopic (-3 D) defocus (1 x 60 minutes), (B) continuous myopic (+3 D) defocus (1 x 60 minutes), (C) alternating low frequency cycles of hyperopic then myopic defocus (2 x 30 minutes), (D) alternating low frequency cycles of myopic then hyperopic defocus (2 x 30 minutes), (E) alternating high frequency cycles of hyperopic then myopic defocus (4 x 15 minutes), and (F) alternating high frequency cycles of myopic then hyperopic defocus (4 x 15 minutes).

During the “defocus period”, each subjects’ right eye was exposed to six different defocus conditions (monocular defocus over the right eye’s optimal distance refraction), while watching movies on a TV at 6 m, with the left eye optimally corrected for distance viewing to maintain relaxed accommodation. This experimental paradigm has been used previously and allows control of the accommodation response with the non-tested (left) eye, while simultaneously producing defocus conditions in the tested (right) eye [25–30]. Over six separate sessions, the right eye was exposed to 60 minute episodes of either continuous or alternating myopic and hyperopic defocus (+3 DS and -3 DS). During the alternating defocus conditions, the eye was exposed to either 30 minutes or 15 minutes of alternating cycles of myopic and hyperopic defocus, with the defocus order reversed in separate sessions (Fig 1).

**Fig 2:**
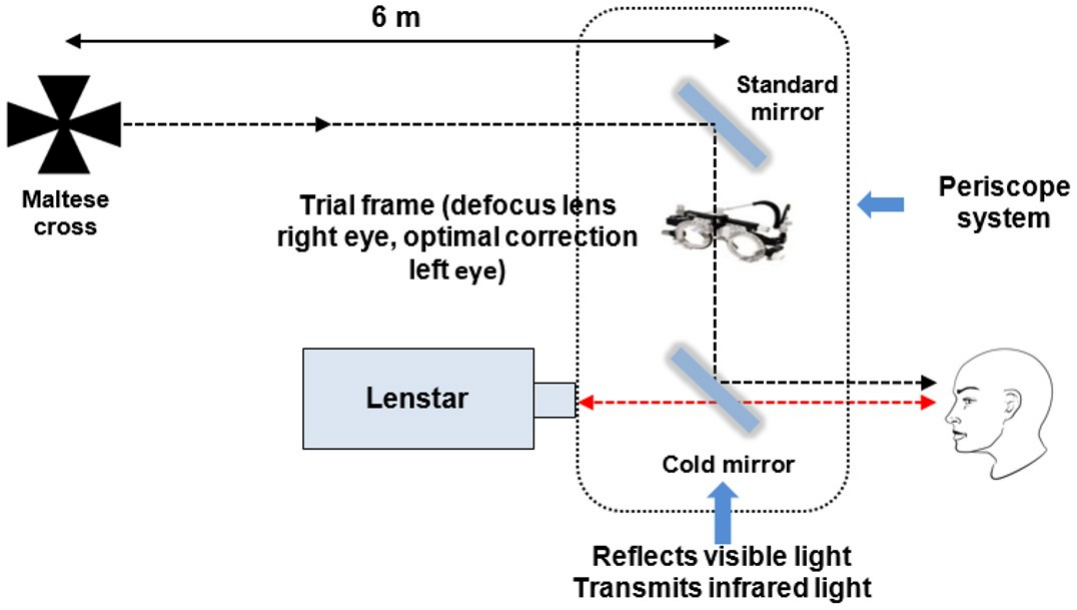
Schematic representation of the experimental set-up for axial length measures. A binocular periscope system was used to allow fixation of the distant target (Maltese cross) while the Lenstar biometer measured axial length.

Five repeated measures of axial length (measured from the anterior corneal surface to the retinal pigment epithelium [RPE]) were obtained at baseline, and then every 15 minutes during both continuous and alternating defocus conditions, using the Lenstar optical biometer (LS 900, Haag Streit AG, Koeniz, Switzerland). This is a non-contact biometry device that works on the principle of optical low-coherence reflectometry (OLCR), using a broad-band light source (range of 20–30 nm), with a central wavelength of 820 nm. The Lenstar optical biometer has a display resolution of 10 μm. To improve the accuracy of the measurements of axial length, only five consecutive measurements with a cumulative standard deviation (as displayed on the Lenstar) of ≤ 7 μm were included. If any of the five consecutive measurements yielded a cumulative SD of > 7 μm, that measurement was deleted and then immediately repeated with a new measurement. To control for accommodation and to provide continuous exposure to defocus during biometry, a binocular beam splitter periscope system was used to allow fixation of an external target (a high contrast Maltese cross) at a 6 m distance during measurement acquisition. In order to provide the periscopic view of the Maltese cross, the system was adjusted and once the centre of the Maltese cross was aligned with the internal fixation target of the biometer (the red fixation target of the Lenstar), the subject was asked to fixate the centre of the target at a 6 m distance. When using the periscope system, the subject’s sphero-cylinder distance refraction was mounted in a trial frame in front of each eye and the additional defocus lens was positioned in front of the right eye. A schematic diagram of this experimental set-up is shown in Fig 2.

To assess the intrasession repeatability of axial length measurements, the within-session standard deviation (SD) and within-session range of five consecutive measurements of axial length across all subjects and all defocus conditions were calculated [51]. The intraclass correlation coefficient (ICC) was also calculated. The within-session SD was 5 μm, with a within-session range of 11 μm, and ICC of 0.999, indicating highly precise measurements of axial length.

The subfoveal choroidal thickness of the right eye (defocused eye) was also measured using spectral domain optical coherence tomography (SD-OCT) (Copernicus SOCT-HR; Optopol Technology SA, Zawiercie, Poland). This device provides high resolution, cross-sectional images of the posterior eye, using a peak wavelength of 850 nm and has axial and transverse resolutions of 3 μm, and 5 μm (in tissue), respectively. The measurements of choroidal thickness were obtained at baseline, and then following removal of the defocus lens at the end of the 60 minute defocus period (immediately following the final measurement of axial length). The OCT images were obtained with a 5 mm horizontal foveal line scan, consisting of 40 B-scans, each with 1500 A-scans, with an acquisition time of 1.52 seconds.

Following data collection, the five repeated measures of axial length at each measurement time point were averaged for each defocus condition, and across subjects. For the measurements of subfoveal choroidal thickness, the raw OCT images were extracted from the instrument and then analysed using custom written software [52]. For each OCT image, the software aligned and averaged the 40 individual B-scans, to generate a high-quality average B-scan image with reduced speckle noise and increased visibility of the posterior segment structures. The resolution of the final OCT images was 2.26 microns per pixel. Each averaged OCT image for each subject at each measurement time point was then manually segmented by an experienced observer who was masked to the time of the measurement and type of defocus condition, for all of the scans.

To provide an assessment of the repeatability and reliability of the subfoveal choroidal thickness segmentation, the masked observer manually segmented the baseline subfoveal choroidal thickness of all subjects for two randomly selected defocus conditions, twice. The coefficient of repeatability and 95% confidence interval of the coefficient of repeatability (derived from both defocus conditions) [51], were 6 μm and 3–9 μm, respectively. A paired sample t-test revealed no significant difference between the two measurements (p > 0.05). Bland-Altman analysis [53] revealed excellent agreement between the two measures of the subfoveal choroidal thickness, with a negligible mean difference of -0.5 μm and 95% limits of agreement of -9 to +7 μm. The intraobserver reliability was assessed using the ICC (two-way mixed model, absolute agreement) and was excellent at 0.999, with a 95% confidence interval of 0.998–1.00.

The Shapiro-Wilk test of normality revealed that the axial length and subfoveal choroidal thickness data did not significantly depart from a normal distribution (p > 0.05). In order to assess the effects of defocus condition for each variable, the data were analysed using a repeated measures ANOVA with two within-subjects factors (time and type of defocus). Following the repeated measures ANOVA, for any variables with significant main effects and interactions (p < 0.05), pairwise comparisons with Bonferroni corrections were conducted. To assess the effects of defocus order and defocus frequency on each variable, a multifactorial linear mixed model analysis was used.

## Results

### Axial length

Repeated measures ANOVA revealed a highly significant interaction between the type of defocus and time of measurement for axial length measures across the six defocus conditions (F _(20,300)_ = 4.60, p < 0.0001).

#### Continuous defocus

**Fig 3:**
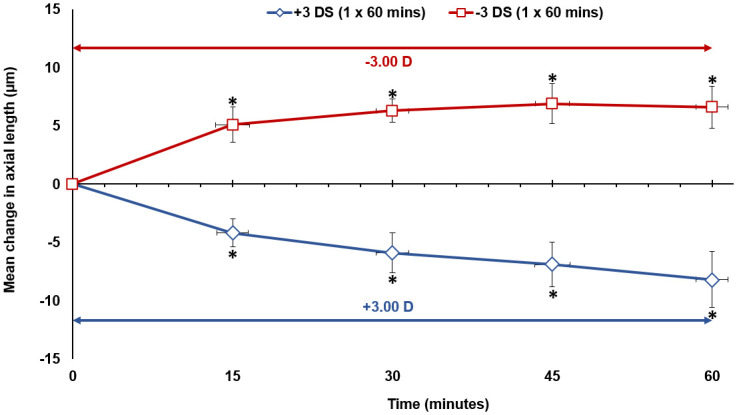
The mean change in axial length from baseline (0 minute time point), during 60 minutes of continuous hyperopic defocus (Condition A) and continuous myopic defocus (Condition B) for all subjects. Vertical error bars represent the standard error of the mean difference in axial length, and horizontal error bars represent the standard error in measurement time. Asterisks indicate a significant mean difference from baseline axial length (p < 0.05).

Pairwise comparisons with Bonferroni corrections revealed that 15 minutes after the introduction of hyperopic defocus (Condition A), there was a significant axial elongation of +5 ± 6 μm from baseline (p = 0.038). Following this initial response, the axial length remained relatively stable until the end of the defocus period and was significantly different from baseline at all subsequent measurement times (all p < 0.05). The maximum axial elongation occurred at 45 minutes with the eye being +7 ± 7 μm longer than the baseline measurement (p = 0.010). The axial length at the end of 60 minutes was +7 ± 7 μm longer than the baseline measurement (p = 0.027) (Table 1) (Fig 3). The introduction of myopic defocus (Condition B) produced a significant axial length reduction of -4 ± 5 μm from baseline after 15 minutes (p = 0.040). The axial length then continued to shorten gradually over the next 45 minutes, reaching a significant maximum axial length reduction of -8 ± 10 μm at the end of the 60 minutes (p = 0.046) (Table 1) (Fig 3).

**Table 1:** Mean change in axial length from baseline across different measurement times for continuous and alternating defocus conditions in all subjects.

	15-minute		30-minute		45-minute		60-minute	
Defocus condition	Mean change ± SD (μm)	P value	Mean change ± SD (μm)	P value	Mean change ± SD (μm)	P value	Mean change ± SD (μm)	P value
Condition A	+5 ± 6	0.038*	+6 ± 4	0.0001*	+7 ± 7	0.010*	+7 ± 7	0.027*
Condition B	-4 ± 5	0.040*	-6 ± 7	0.043*	-7 ± 7	0.025*	-8 ± 10	0.046*
Condition C	+5 ± 8	0.219	+7 ± 9	0.045*	+1 ± 12	1.000	0 ± 11	1.000
Condition D	-4 ± 7	0.345	-6 ± 9	0.115	0 ± 11	1.000	-2 ± 12	1.000
Condition E	+5 ± 8	0.330	-1 ± 9	1.000	-2 ± 9	1.000	-4 ± 7	0.251
Condition F	-3 ± 6	0.307	-2 ± 7	1.000	-4 ± 6	0.265	-3 ± 8	1.000

Asterisk * indicates a significant change (p < 0.05) in axial length from the baseline measurement.

#### Alternating defocus (30 minute cycles)

**Fig 4:**
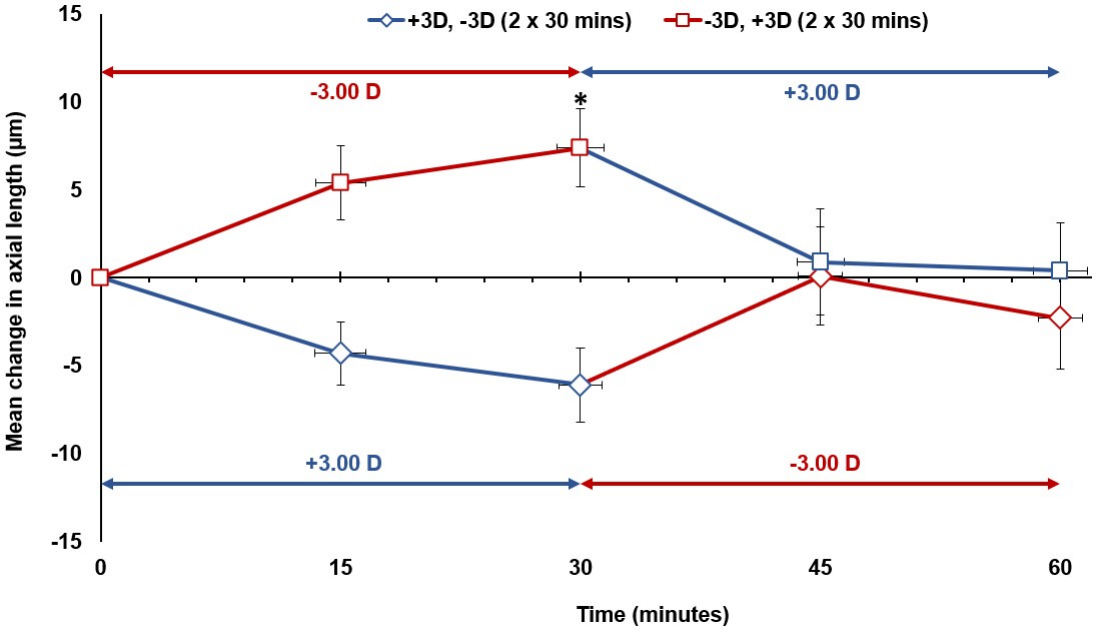
The mean change in axial length from baseline (0 minute time point) during 30 minute alternating cycles of hyperopic then myopic defocus (Condition C), and myopic then hyperopic defocus (Condition D) for all subjects. Vertical error bars represent the standard error of the mean difference in axial length, and horizontal error bars represent the standard error in measurement time. Asterisks indicate a significant mean difference from the baseline axial length (p < 0.05).

When the right eye was exposed to 30 minute alternating cycles of hyperopic then myopic defocus (Condition C), after the first 30 minutes of hyperopic defocus, the axial length was significantly longer than baseline by +7 ± 9 μm (p = 0.045). The introduction of myopic defocus then gradually cancelled the previous axial elongation effects of hyperopic defocus and over the next 30 minutes the eye approached the baseline axial length level (mean difference of 0 ± 11 μm from baseline at the end of the 60 minutes, p > 0.05) (Table 1) (Fig 4). A similar cancellation effect of opposing blur was observed when the right eye was exposed to 30 minute alternating cycles of myopic then hyperopic defocus (Condition D). After the first 30 minutes of myopic defocus, the axial length was shorter than the baseline by -6 ± 9 μm (p > 0.05). This reduction in axial length then gradually cancelled during the next 30 minutes of hyperopic defocus of equal power, and by the end of 60 minutes, the eye was almost at the baseline level (mean difference of -2 ± 12 μm, p > 0.05) (Table 1) (Fig 4).

#### Alternating defocus (15 minute cycles)

**Fig 5:**
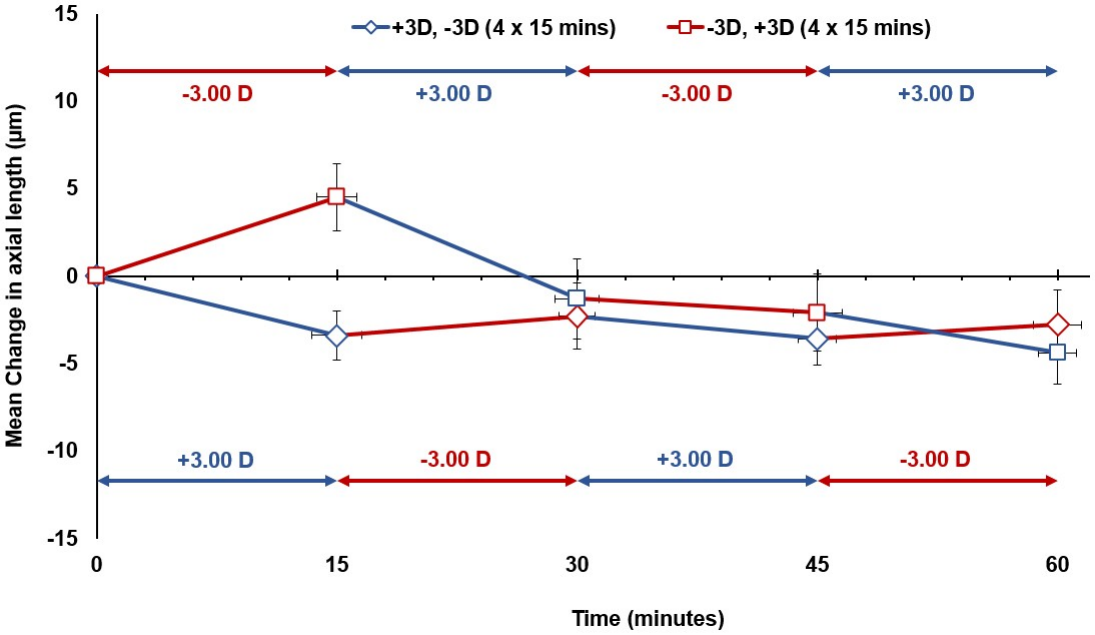
The mean change in axial length from baseline (0 minute time point) during 15 minute alternating cycles of hyperopic then myopic defocus (Condition E), and myopic then hyperopic defocus (Condition F) for all subjects. Vertical error bars represent the standard error of the mean difference in axial length, and horizontal error bars represent the standard error in measurement time. Asterisks indicate a significant mean difference from the baseline axial length (p < 0.05).

When the eye was exposed to 15 minute alternating cycles of hyperopic and myopic defocus, a cancellation effect of opposing blur sessions did not occur as the axial length was observed to shorten slowly over time. During 60 minute alternating cycles of hyperopic then myopic defocus (Condition E), after the first 15 minutes of uninterrupted hyperopic defocus, axial length changed by +5 ± 8 μm from baseline (p > 0.05). This increase in axial length was then cancelled during the next 15 minutes of uninterrupted myopic defocus, as the eye returned to below baseline level at 30 minutes. From 30 to 60 minutes, axial length continued to shorten slowly and reached a -4 ± 7 μm reduction from baseline following the final defocus session (p > 0.05) (Table 1) (Fig 5). A similar pattern of response was also observed for axial length during 15 minute alternating cycles of myopic then hyperopic defocus (Condition F), as it shortened gradually over time. Following completion of the two cycles of myopic then hyperopic defocus, the axial length was -3 ± 8 μm less than the baseline measurement at 60 minutes (p > 0.05) (Table 1) (Fig 5).

#### Effects of defocus order and defocus frequency

Assessment of the effect of defocus order revealed that regardless of the frequency of the cycles, there was no significant difference in the final axial length response to defocus observed when the eye was exposed to myopic then hyperopic defocus, or hyperopic then myopic defocus (p > 0.05). Similarly, there was no significant difference in the final axial length response between the 15 minute and 30 minute cycling frequencies for each order of defocus, indicating no significant effects of defocus frequency (p > 0.05).

### Axial length changes: Continuous vs alternating defocus

After the first 30 minutes of uninterrupted hyperopic defocus, baseline axial length increased by a similar magnitude during both the continuous hyperopic defocus (Condition A) and the alternating hyperopic then myopic defocus (Condition C) conditions (+6 ± 4 μm vs +7 ± 9 μm, p > 0.05, Figs 3 and 4 respectively). From 30 minutes to 60 minutes, axial length remained stable during continuous hyperopic defocus (+1 ± 6 μm increase, p > 0.05, Fig 3), while it reduced significantly by -7 ± 10 μm during alternating hyperopic then myopic defocus (p = 0.022, Fig 4). The magnitude of change in axial length from 30 minutes to 60 minutes between the continuous hyperopic defocus and the alternating hyperopic then myopic defocus conditions was significantly different (p = 0.033).

After the first 30 minutes of uninterrupted myopic defocus, axial length decreased by -6 ± 7 μm during continuous myopic defocus (Condition B) and by -6 ± 9 μm during the alternating myopic then hyperopic defocus condition (Condition D) (p > 0.05, Figs 3 and 4). From 30 minutes to 60 minutes, axial length changed by -2 ± 6 μm during continuous myopic defocus (p > 0.05, Fig 3) and by +4 ± 12 μm during the alternating myopic then hyperopic defocus condition (p > 0.05, Fig 4). There was a significant difference in the magnitude of change in axial length from 30 minutes to 60 minutes between the continuous myopic defocus and the alternating myopic then hyperopic defocus conditions (p = 0.044).

After the first 15 minutes of uninterrupted hyperopic defocus, baseline axial length increased significantly and by a similar magnitude during both the continuous hyperopic defocus (Condition A) and the alternating hyperopic then myopic defocus (Condition E) conditions (+5 ± 6 μm vs +5 ± 8 μm, p > 0.05, Figs 3 and 5 respectively). From 15 minutes to 60 minutes, axial length remained relatively stable during continuous hyperopic defocus (mean change of +2 ± 7 μm p > 0.05, Fig 3) while it shortened significantly by -9 ± 8 μm (p < 0.0001, Fig 5) during the alternating hyperopic then myopic defocus condition.

After the first 15 minutes of uninterrupted myopic defocus, axial length changed by -4 ± 5 μm during continuous myopic defocus (Condition B) and by -3 ± 6 μm during alternating myopic then hyperopic defocus condition (Condition F) (p > 0.05, Figs 3 and 5). From 15 minutes to 60 minutes, axial length changed by -4 ± 8 μm during continuous myopic defocus (p = 0.05, Fig 3) while it remained stable during alternating myopic then hyperopic defocus condition (+1 ± 9 μm, p > 0.05, Fig 5).

### Subfoveal choroidal thickness

Imposing defocus resulted in some significant changes in subfoveal choroid thickness over time. At 60 minutes, the subfoveal choroid was -4 ± 11 μm thinner than baseline after continuous hyperopic defocus (Condition A) (p > 0.05), while it was significantly thicker by +8 ± 11 μm following continuous myopic defocus (Condition B) (p = 0.030) (Table 2). When exposed to 30 minute or 15 minute alternating cycles of defocus, subfoveal choroidal thickness did not differ significantly from baseline after hyperopic then myopic defocus (Condition C & Condition E), or after myopic then hyperopic defocus (Condition D & Condition F) (p > 0.05) (Table 2). There were no significant effects of defocus order and defocus frequency for any of the analyses of the subfoveal choroidal thickness measures (all p > 0.05).

**Table 2:** Mean change in subfoveal choroidal thickness from baseline across different defocus conditions in all subjects.

	Condition A	Condition B	Condition C	Condition D	Condition E	Condition F
Mean change ± SD (μm)	-4 ± 11	+8 ± 11	+7 ± 14	+2 ± 15	+5 ± 17	+5 ± 17
P value	0.208	0.028*	0.087	0.685	0.278	0.273

Asterisk * indicates a significant change (p < 0.05) in baseline subfoveal choroidal thickness after 60 minutes of defocus exposure.

## Discussion

This study has shown for the first time that the human eye is capable of temporal averaging of visual cues from alternating myopic and hyperopic defocus. Whilst exposure to continuous defocus led to significant bi-directional changes in axial length, the change in axial length was minimal when the eye was exposed to alternating periods of myopic and hyperopic defocus. During a 30 minute cycling frequency, alternating episodes of defocus of opposite power largely cancelled each other, and the eye remained at near baseline levels after 60 minutes. During a 15 minute cycling frequency, the eye’s response to myopic defocus appeared to be greater than the response to hyperopic defocus, as the eye was slightly shorter than the baseline measurement at 60 minutes, however this change was not statistically significant. Similar but smaller magnitude changes were also observed for subfoveal choroidal thickness.

The findings from the continuous defocus conditions are consistent with those from previous studies of human [26, 29–31] and other animal eyes [6, 7, 36] where small but significant changes in axial length and choroidal thickness have been reported after short term exposure to continuous myopic and hyperopic defocus. A recent investigation in human eyes has shown a significant change in axial length, approximately 2 minutes after exposure to imposed defocus [26]. Similarly, significant bi-directional changes in subfoveal choroidal thickness after 10 to 35 minutes have been reported [30]. An investigation in school children found no significant change in axial length after 60 minutes exposure to defocus [25], however, as the measurements were taken following the instillation of 1% cyclopentolate (an antimuscarinic agent that is known to affect the thickness of the choroid and axial length) [54, 55], these findings may have been influenced by the effects of the drug. Further, in a recent investigation, a 30 minute exposure to myopic defocus with full field and multifocal contact lenses yielded no significant change in choroidal thickness [56]. This difference in outcomes may be due to the shorter duration of exposure to defocus or to the influence of the contact lens correction. It is evident from our findings during the continuous defocus conditions that the human eye is able to respond to the sign of blur rapidly (within 15 minutes) and make distinct bi-directional changes in its axial length. Although the underlying mechanisms of this response are not fully understood; a variety of potential mechanisms such as the role of contrast cues from contrast adaptation (changes in contrast sensitivity at different spatial frequencies) [57–60], colour cues from chromatic aberration [61–64], or optical vergence cues from image defocus [65] have all been suggested as potential ways in which the human eye decodes the sign of blur.

The maximum mean axial length change observed during continuous hyperopic defocus was +7 ± 7 μm of elongation and during continuous myopic defocus was a -8 ± 10 μm reduction. These findings correspond closely with published data in human eyes where changes of similar magnitude in axial length following continuous short term exposure to myopic and hyperopic defocus have been reported [26, 29]. However, when the pattern of defocus was alternated at a 30 minute frequency, the eye underwent a significantly different temporal pattern of change than it did during continuous defocus. The axial length response to each type of defocus after 30 minutes was almost cancelled during the succeeding 30 minute exposure to defocus of opposite power, and the axial length was near baseline levels after 60 minutes. This finding occurred irrespective of the order of defocus, indicating that the cancelling effects of myopic and hyperopic defocus for this particular duration and alternating frequency were of similar strength.

When the frequency of alternating defocus cycles was 15 minutes, the myopic and hyperopic defocus did not appear to completely cancel the preceding effects of each other; rather, a slight non-significant trend towards a more dominant response to myopic defocus was observed, and axial length was observed to reduce slightly. In animal models of blur integration, a greater potency of myopic defocus in arresting the ocular elongation effect of hyperopic defocus has been reported. For instance, when myopic and hyperopic defocus were presented sequentially or simultaneously in chicks [6, 36–39], tree shrews [40, 41], monkeys [42], and marmosets [43], the eye responded preferentially to myopic defocus and developed less myopia/more hyperopia. Even when episodes of myopic and hyperopic defocus had equal duration when imposed alternatively over the chick eye, the axial growth still reduced, suggesting that myopic defocus provides a stronger growth signal [37]. Since the slight bias towards the dominating effects of myopic defocus observed in our study was not statistically significant, we cannot conclude if such properties also exist in the human eye. Future investigations involving longer durations of exposure to defocus or utilizing different magnitudes of defocus may provide additional insights into the relative influence of myopic versus hyperopic blur.

Defocus-mediated changes in axial length are expected to occur through modulations in the thickness of the choroid posterior to the retina, and thus affects the measurements of the axial length to the overlying RPE [26–30]. We found the bi-directional changes in the thickness of the subfoveal choroid to be consistent with the direction of the observed changes in axial length at the end of each defocus condition. In general, subfoveal choroidal thickening occurred along with axial length reduction, and subfoveal choroidal thinning occurred along with axial elongation. However, for the continuous defocus conditions, subfoveal choroidal thickening accounted for 87% of the observed mean axial length reduction during myopic defocus, and only accounted for 57% of the observed mean axial elongation during hyperopic defocus. A similar pattern of response was also observed for axial length and subfoveal choroidal thickness during alternating defocus. The discrepancies in the magnitude of axial length and choroidal thickness changes could have arisen in two possible ways. First, the final measurement of the subfoveal choroidal thickness was obtained approximately one minute after the removal of the defocus lens, therefore, during this brief period of clear viewing during the OCT imaging process, some decay in the effects of defocus could have occurred. Alternately, other factors such as expansion or contraction of the sclera might have also contributed to the observed axial length elongation and reduction with defocus. Since current imaging technology does not allow visualization of the thickness of the posterior sclera in most eyes, future studies to understand the potential role of the sclera in mediating the human eye’s response to short-term defocus seem warranted.

The changes in choroidal thickness with defocus (thickening or thinning) were found to range between 4 to 8 μm across the different defocus conditions investigated in this study. Whilst this amount of change is above the axial resolution of the OCT device (3 μm in tissue), it is within the range of the coefficient of repeatability and its 95% confidence interval for the measurements of choroidal thickness in this study (6 μm and 3–9 μm, respectively). Therefore, this limitation should be noted when considering the choroidal thickness changes observed in this study.

Previous studies suggest two potential models in which defocus signals could be integrated over time; a simple linear model, and a more complex non-linear model [6, 35, 37, 66, 67]. Based on a simple linear model, the effects of equal powers of defocus of opposite sign would be added linearly so that the resulting compensation would be relative to the average exposure of defocus that the retina experienced, summed over a period of time (e.g. +3 D for 30 minutes followed by -3 D for 30 minutes = 0 change). However, based on a non-linear model, more complex outcomes of blur integration are expected where the final compensation could be multifactorial, depending on the sign and power of the defocus experienced (myopic or hyperopic blur), and the frequency and duration of individual episodes of blur. Whilst our findings from the alternating defocus conditions suggest a simple summation of the effects of defocus of opposing sign over time, the findings from our continuous defocus conditions do not reflect a simple linear model. We observed that the rate of axial elongation and axial length reduction resulting from exposure to equal amounts of hyperopic and myopic defocus was not linearly proportional to the time exposed to blur. During continuous defocus exposure, almost 70% of the final axial elongation observed in response to hyperopic defocus (+7 ± 7 μm) occurred after 15 minutes of exposure to defocus, and 50% of the final axial length reduction (-8 ± 10 μm) in response to myopic defocus occurred after 15 minutes of exposure to defocus, indicating that the time course of the eye’s response to blur may be non-linear and may vary depending on the sign of defocus. Whilst experiments with animals have shown characteristics which strongly suggest a complex, non-linear model of temporal blur integration [6, 36–44] the mixed findings from our investigations do not allow us to confidently propose the model of temporal blur integration in human eyes. Further studies in this field seem warranted.

It has been proposed that the transient exposure to hyperopic defocus associated with near activities (e.g. due to lag of accommodation) [68–71], ocular aberrations [72, 73] or peripheral defocus [74] might predispose the eye to myopia. We found that exposure to myopic defocus is able to quickly counterbalance the axial elongation and subfoveal choroidal thinning effects of hyperopic defocus. If short-term ocular changes in response to defocus are associated with longer term refractive error development in the human eye, then optical methods of introducing myopic blur for at least a similar duration to that of exposure to hyperopic blur, may counteract the myopigenic stimulus. However, it must be noted that only one level of myopic and hyperopic defocus and limited alternating defocus frequencies were tested in this study.

The inter-subject variability in the axial length and choroidal thickness response to blur observed in this study could have been due to individual differences in retinal sensitivity to blur (e.g. an equal level of defocus degrades visual acuity by varying amounts between individuals). Also, while we attempted to control many of the known factors that influence choroidal thickness and axial length (diurnal rhythms, near work, medications, smoking, caffeine intake, and dynamic exercise) there are many other systemic factors that could potentially vary from day to day and affect the highly vascularized and sympathetically innervated choroid.

In conclusion we have shown for the first time that the human eye is capable of temporal integration of myopic and hyperopic defocus signals. Over 60 minutes of blur exposure, this integration was effectively a simple summation (cancellation) of the effects of the preceding opposite sign blur condition.

## Supporting information

S1 Dataset Ocular biometry data for each of the defocus conditions for all subjects enrolled in the study. (XLSX)
